# Comprehensive Profiling of Serum Exosomes by a Multi-Omics Approach Reveals Potential Diagnostic Markers for Brain Metastasis in Lung Cancer

**DOI:** 10.3390/cancers17121929

**Published:** 2025-06-10

**Authors:** Jiwoo Lim, Mia Kang, Young-Ho Ahn, Min-Sun Cho, Jin Hwa Lee, Jihee Lee Kang, Youn-Hee Choi

**Affiliations:** 1 Department of Physiology, College of Medicine, Ewha Womans University, Seoul 07804, Republic of Korea; jwl@ewha.ac.kr (J.L.); memoria0110@ewhain.net (M.K.); jihee@ewha.ac.kr (J.L.K.); 2 Inflammation-Cancer Microenvironment Research Center, College of Medicine, Ewha Womans University, Seoul 07804, Republic of Korea; yahn@ewha.ac.kr (Y.-H.A.); jinhwalee@ewha.ac.kr (J.H.L.); 3 Department of Molecular Medicine, College of Medicine, Ewha Womans University, Seoul 07804, Republic of Korea; 4 Department of Pathology, College of Medicine, Ewha Womans University, Seoul 07804, Republic of Korea; mcho1124@ewha.ac.kr; 5 Division of Pulmonary and Critical Care Medicine, Department of Internal Medicine, College of Medicine, Ewha Womans University, Seoul 07804, Republic of Korea

**Keywords:** lung cancer, brain metastasis, exosomes, miR-206-3p, vinculin

## Abstract

Background: Brain metastasis occurs in 40–50% of lung cancer patients and is associated with poor prognosis. This study aimed to identify potential exosomal biomarkers for the early detection of brain metastasis in lung cancer using a comprehensive multi-omics approach. Methods: Using a lung cancer mouse model, which develops brain metastasis, we collected serum samples at different stages (control, 6 weeks for lung cancer, and 10 weeks for brain metastasis). We profiled the contents of serum-derived exosomes using small RNA sequencing and LC-MS/MS proteomic analysis, and assessed the clinical relevance of candidate biomarkers using publicly available patient datasets. Results: RNA sequencing identified 11 differentially expressed miRNAs across disease progression, with miR-206-3p showing significant upregulation during brain metastasis. Pathway analysis of miR-206-3p targets revealed enrichment in cancer-related pathways, including Hippo, MAPK, Ras, and PI3K-Akt signaling. Proteomic analysis revealed 77 proteins specifically upregulated in the brain metastasis stage, with vinculin (VCL) emerging as a promising marker. While VCL expression decreased in lung tissues and showed no significant changes in brain tissues, its levels were significantly elevated in serum-derived exosomes during brain metastasis. Clinical database analysis revealed that higher VCL expression correlated with poor patient survival. Conclusions: Our study identified exosomal miR-206-3p and VCL as promising non-invasive biomarkers for brain metastasis in lung cancer using the mouse model. These findings provide new opportunities for early detection and monitoring of brain metastasis, potentially enabling timely therapeutic intervention.

## Simple Summary

Brain metastasis frequently occurs in lung cancer patients and is associated with a poor prognosis, often due to late detection. Existing imaging methods have limitations for early diagnosis. This study aimed to identify non-invasive biomarkers using serum-derived exosomes from a lung cancer brain metastasis mouse model. Through comprehensive multi-omics analysis of exosomal contents, we identified miR-206-3p and vinculin (VCL) as potential biomarker candidates specifically linked to brain metastasis. Exosomal miR-206-3p showed significant upregulation during brain metastasis. Exosomal VCL levels were notably elevated at this stage, and higher VCL correlated with poor overall survival in clinical data. These findings suggest that these exosomal molecules represent promising non-invasive diagnostic tools that may facilitate earlier detection of brain metastasis in lung cancer. Moreover, our model may serve as a valuable platform for conducting functional studies and investigating their roles in promoting brain metastatic progression.

## 1. Introduction

Brain metastasis occurs when tumor cells from a primary tumor or secondary metastases outside the central nervous system spread through the bloodstream and form an intracranial tumor seeding in the brain’s microvasculature [1,2]. Any type of cancer can lead to brain metastasis, but in adults, lung, breast, and melanoma are the primary cancers most likely to spread to the brain [2,3]. Brain metastasis occurs in various cancers, including lung, breast, and colorectal cancers, and is associated with a poor prognosis [4,5]. Lung cancer accounts for 40–50% of all brain metastasis cases [6,7]. Currently, brain metastases are typically diagnosed after patients present with neurological symptoms, and even with subsequent radiotherapy or surgery, the prognosis remains poor [8,9]. A significant limitation in current cancer treatment is the lack of reliable methods to identify patients at risk of developing brain metastases, preventing early detection and intervention due to the absence of predictive biomarkers. Therefore, the development of biomarkers for early diagnosis is crucial and could significantly improve patient survival rates.

Exosomes, membrane-bound extracellular vesicles measuring 40–150 nm that are secreted by all cells, carry molecular cargo including DNA, RNA, and proteins within their lipid bilayer [10,11]. Exosomes are found in the tumor microenvironment, and growing evidence indicates that they contribute to tumor development by influencing angiogenesis, immune responses, and metastasis [12,13]. Given their crucial role in intercellular communication and their ability to reflect the pathological state of their parent cells, exosomes have recently garnered considerable attention as potential circulating biomarkers due to their several advantageous properties. Firstly, body fluids like blood, urine, and saliva contain abundant exosomes, making them easily accessible for non-invasive liquid biopsy [14,15]. Secondly, cancer-derived exosomes carry functional molecular cargo that closely reflects the genetic and molecular composition of their cells of origin [16,17]. The proteins, nucleic acids, and lipids encapsulated in exosomes provide a snapshot of the tumor’s biological state and can offer insights into cancer-specific alterations. Thirdly, the lipid bilayer of exosomes protects their molecular contents from degradation, preserving the biological activity and stability of potential biomarkers [18].

Several studies have demonstrated the utility of cancer-derived exosomes as diagnostic and prognostic markers across various cancer types. For example, exosomal glypican-1 serves as a highly specific early detection marker in pancreatic cancer, while exosomal miR-21 and miR-105 have been identified in breast cancer as promising markers that correlate with disease progression and metastasis [19,20]. In lung cancer, a recent comparative proteomic analysis of plasma exosomes from patients with liver and brain metastases identified candidate proteins differentially enriched in brain metastatic cases, suggesting the potential of exosome-based biomarkers for organ-specific metastatic patterns [21]. While these studies highlight the diagnostic value of exosomal biomarkers, they primarily reflect cross-sectional analyses of advanced metastatic disease.

In this study, we aimed to identify and validate serum exosome-derived miRNA and protein biomarkers associated with the development of brain metastasis in lung cancer. To this end, we conducted a longitudinal multi-omics analysis of serum-derived exosomes using a well-characterized mouse model of lung cancer brain metastasis. By integrating RNA sequencing, proteomic profiling, and bioinformatic analyses, we investigated exosomal miRNA and protein signatures across different stages of disease progression. Notably, miR-206-3p and vinculin (VCL) emerged as promising biomarker candidates, both showing expression patterns correlated with metastatic development. These findings may ultimately contribute to the development of non-invasive tools for early detection and monitoring of brain metastasis in lung cancer.

## 2. Materials and Methods

### 2.1. Cell Culture

The murine lung cancer cell line 344SQ-RFP was kindly provided by Prof. Young-Ho Ahn (Ewha Womans University, Seoul, Republic of Korea). Cells were cultured in RPMI 1640 medium (Welgene, Gyeongsan, Republic of Korea) supplemented with 10% fetal bovine serum (FBS; Thermo Scientific, Waltham, MA, USA) and 1% penicillin/streptomycin (100 U/mL penicillin, 100 μg/mL streptomycin; Welgene) at 37 °C in a humidified incubator with 5% CO_2_. The Vcl-specific siRNAs (Bionics, Seoul, Republic of Korea) were transiently transfected into 344SQ-RFP cells using Lipofectamine 3000 Transfection Reagent (Thermo Scientific) according to the manufacturer’s instructions.

### 2.2. Mouse Experiments

All mouse experiments were approved by the Institutional Animal Care and Use Committee (IACUC) of Ewha Womans University College of Medicine (EWHA MEDIACUC 21-036). Mice were cared for and euthanized according to IACUC standards. Syngeneic (129/Sv) mice at 8 weeks were subcutaneously injected with 344SQ-RFP cells (1 × 10^6^ cells/mouse) in the right flank. Each experimental group (control, 6-week, and 10-week) initially consisted of 10 mice. Mice were monitored twice a week for tumor growth and survival, and were euthanized at 6 weeks and 10 weeks [22]. Due to tumor progression, not all mice survived to the designated endpoints; analyses were performed using viable mice at each time point. Whole blood was collected, and serum was frozen at −80 °C until exosome collection.

### 2.3. H&E Staining

For histological analysis, brain tissues were fixed in 4% paraformaldehyde for 24 h at 4 °C and embedded in paraffin. The paraffin blocks were sectioned at 4 μm thickness using a microtome. The sections were deparaffinized in xylene and rehydrated through a graded series of ethanol solutions (100%, 95%, 80%, and 70%). After washing with distilled water, the sections were stained with Harris hematoxylin solution for 5 min, followed by washing under running tap water for 5 min. The sections were then differentiated in 1% acid alcohol for 30 s and washed again. After counterstaining with eosin solution for 30 s, the sections were dehydrated through graded ethanol solutions (70%, 80%, 95%, and 100%) and cleared in xylene. Finally, the sections were mounted using mounting medium (Thermo Scientific) and observed under a light microscope.

### 2.4. Exosome Isolation

Exosomes were isolated from serum using ExoQuick (System BioSciences, Palo Alto, CA, USA) following the manufacturer’s instructions. Briefly, ExoQuick solution was added to the serum at a ratio of 1:4 (ExoQuick to serum), and the mixture was incubated at 4 °C for 30 min. After incubation, the samples were centrifuged at 1500× *g* for 30 min to pellet the exosome fraction. The pellet was resuspended in 100 μL of either PBS or RIPA buffer for downstream analyses.

### 2.5. Nanoparticle Tracking Analysis (NTA)

Nanoparticle tracking analysis (NTA) was performed using NS300 (Malvern Panalytical, Malvern, UK) according to the manufacturer’s instructions. Samples were analyzed at a camera level of 15 and a detection threshold of 3. Exosome concentration was measured with sample dilutions ranging from 1:1000 to 1:10,000 to achieve an optimal particle count of 20 to 100 particles per frame.

### 2.6. Transmission Electron Microscopy (TEM)

Purified exosomes were diluted to 1:10,000 in PBS. Then, 5 μL of the diluted exosome solution was applied to Formvar-carbon-coated electron microscope (EM) grids. The grids were then stained with 2% uranyl acetate, and excess stain was removed with filter paper before air drying. Imaging was performed using H7650 TEM (Hitachi, Tokyo, Japan) at an accelerating voltage of 80 kV.

### 2.7. Western Blot Analysis

Exosome protein concentrations were determined using the BCA Protein Assay Kit (GenDEPOT, Katy, TX, USA). A total of 25 μg of exosomal proteins was separated by SDS-PAGE and transferred onto a PVDF membrane. The membrane was then incubated at 4 °C overnight with primary antibodies specific for CD9 (Cell Signaling Technology, Danvers, MA, USA; #98327), CD63 (Abcam, Cambridge, UK; ab217345), CD81 (Abcam, ab109201), VCL (Cell Signaling Technology; #13901), and β-actin (Santa Cruz Biotechnology, Dallas, TX, USA; sc-69879). After extensive washing, the membrane was incubated with HRP-conjugated secondary antibodies (Cell Signaling Technology). Detection was performed using SuperSignal West Pico Plus Substrate (Thermo Scientific), and the membrane was visualized using an ImageQuant 800 imaging system (Cytiva, Marlborough, MA, USA).

### 2.8. RNA Extraction and RT-qPCR

Total RNA was extracted from tissue samples using the easy-BLUE™ total RNA Extraction Kit (iNtRON, Seongnam, Republic of Korea), according to the manufacturer’s instructions. For mRNA expression analysis, cDNA was synthesized using the ReverTra Ace^®^ qPCR RT Master Mix (TOYOBO, Osaka, Japan), and quantitative PCR was performed using the SYBR™ Green Realtime PCR Master Mix (TOYOBO). mRNA levels were normalized to *Gapdh* expression. For tissue-derived miRNA analysis, total RNA was similarly extracted using the easy-BLUE™ kit, followed by cDNA synthesis and qPCR using the HB miR Multi Assay kit (Heimbiotek, Seongnam, Republic of Korea), in accordance with the manufacturer’s protocol. miRNA expression levels were normalized to *RNU6B* snoRNA levels. For exosomal RNA analysis, total RNA was isolated from purified exosomes using the SeraMir™ Exosome RNA kit (System Biosciences), according to the manufacturer’s instructions. Exosomal miRNA levels were quantified using the HB miR Multi Assay Kit and normalized to miR-16-5p, which served as an internal control [23]. For gene expression analysis following siRNA-mediated knockdown in 344SQ-RFP cells, total RNA was extracted using the easy-BLUE™ kit. cDNA synthesis and qPCR were performed using qPCR RT Master Mix and SYBR Green Realtime PCR Master Mix as described above, and mRNA levels were normalized to *Gapdh* expression. The primer sequences used in this study are listed in Supplementary Table S1.

### 2.9. Small RNA Library Preparation and Sequencing

Small RNA sequencing was performed using pooled samples from three groups: control (*n* = 5), 6-week (*n* = 5), and 10-week (*n* = 5). Next-generation sequencing libraries were prepared using the TailorMix MicroRNA Sample Preparation Version 2 protocol (SeqMatic LLC, Fremont, CA, USA). Briefly, a 3′-adapter was ligated to the RNA samples, and excess 3′-adapters were removed. Subsequently, the 5′-adapter was ligated to the 3′-adapter ligated samples. First-strand cDNA synthesis was then performed, followed by amplification and barcoding of the cDNA library using enrichment PCR. The final RNA library was size-selected using an 8% TBE polyacrylamide gel. Sequencing was carried out on the Illumina NextSeq 500 platform (Illumina, San Diego, CA, USA), with a 1 × 75 bp single-end read configuration.

### 2.10. In Gel Digestion and Liquid Chromatography–Tandem Mass Spectrometry (LC-MS/MS)

Exosome samples were concentrated by ultrafiltration and separated by SDS-PAGE. Gel bands were excised, reduced with 10 mM DTT for 30 min at 60 °C, alkylated with 20 mM iodoacetamide for 45 min at room temperature in the dark, and digested overnight at 37 °C with trypsin (Thermo Scientific, #90058). Peptides were extracted with 5% formic acid and 60% acetonitrile and then dried under vacuum. Peptides were analyzed using a Dionex Ultimate 3000 RLSC Nano System (Thermo Fisher) interfaced with an Eclipse Mass Spectrometer (Thermo Scientific). Samples were loaded onto an Acclaim PepMap 100 trap column (Thermo Scientific) and separated on a NanoEase MZ Peptide BEH C18 column (Waters). Peptides were fractionated at 300 nL/min using a gradient of 4–15% buffer B for 30 min, 15–25% B for 40 min, 25–50% B for 44 min, and 50–90% B for 11 min, then returned to 4% B for 5 min (buffer B: 0.16% formic acid, 80% acetonitrile). DIA workflows were used for data acquisition, with MS scans (400–1200 m/z, resolution 12,000, AGC 3E6) and MS/MS scans (resolution 30,000) at a relative collision energy of 30. Raw data were analyzed using DIA-NN (version 1.8.1) and a predicted peptide library [24].

### 2.11. Gene Ontology, KEGG, and STRING Analysis

Gene Ontology (GO) term and KEGG pathway enrichment analyses were performed using ShinyGO [25,26]. The gene list was input into ShinyGO to identify significant GO terms, categorized into biological processes (BP), molecular function (MF), and cellular components (CC). KEGG pathway enrichment was also conducted to explore associated biological pathways. Protein–protein interaction (PPI) network analysis was performed using the STRING database, and k-means clustering was applied to identify functionally related groups within the PPI network [27].

### 2.12. Gene Expression and Survival Analysis

The GEPIA2 web server was used to analyze VCL expression and its clinical significance in lung adenocarcinoma (LUAD) [28]. Differential expression analysis of VCL between tumor (*n* = 483) and normal tissue samples (*n* = 347) was performed using log2(TPM + 1) transformed expression data. For survival analysis, patients were stratified into quartiles based on VCL expression levels, and the top (Q4) and bottom (Q1) quartiles were compared (*n* = 120 per group). Overall survival was analyzed using the Kaplan–Meier method, and the log-rank test was used to determine statistical significance. The hazard ratio (HR) was calculated using the Cox proportional hazards regression model.

### 2.13. Statistical Analysis

Statistical analyses were conducted using GraphPad Prism version 5 software (GraphPad Software, San Diego, CA, USA). For survival analysis, survival times were recorded for each mouse, and a Kaplan–Meier survival curve was generated. Statistical differences in survival between groups were assessed using the log-rank (Mantel–Cox) test. Comparisons between two groups were performed using a paired Student’s t-test. For comparisons among multiple groups, one-way analysis of variance (ANOVA) followed by Tukey’s post hoc test was used. RT-qPCR and Western blot data are presented as the mean ± standard error of the mean (SEM) from at least three independent experiments. Statistical significance was set as *p* < 0.05.

## 3. Results

### 3.1. Development of a Lung Cancer Brain Metastasis Mouse Model

**Figure 1:**
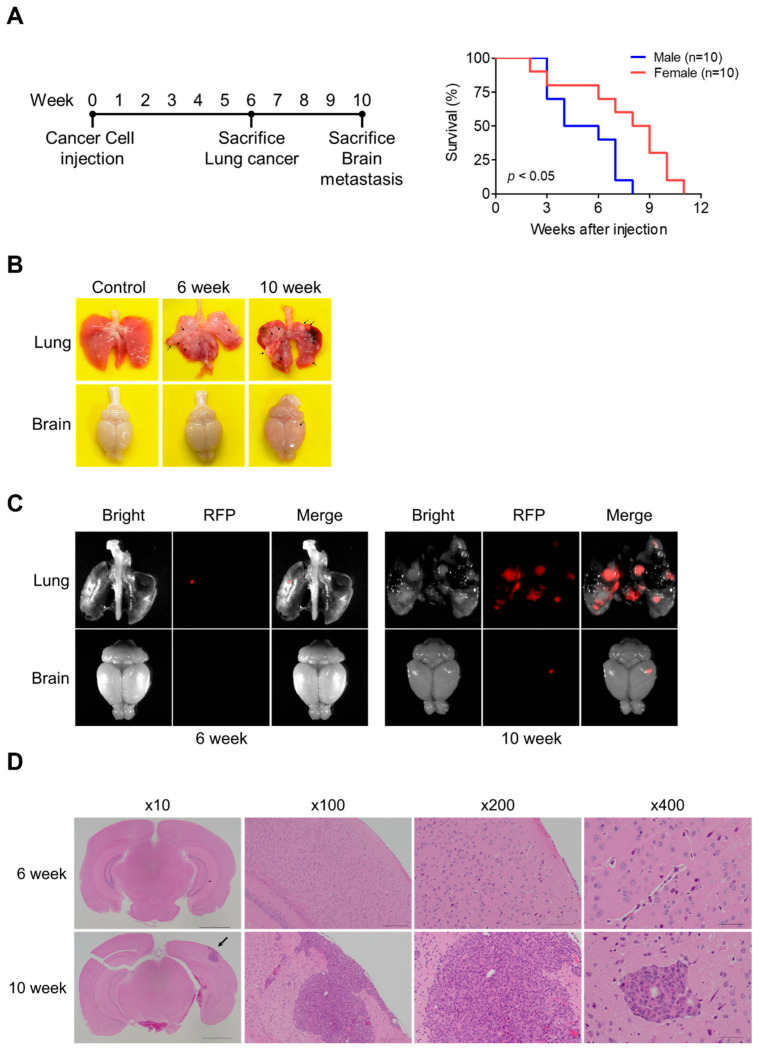
Establishment and characterization of a lung cancer brain metastasis mouse model using 344SQ-RFP cells. (**A**) Left: Experimental timeline showing key events: initial cancer cell injection (0 week), analysis of lung cancer (6 week), and evaluation of brain metastasis (10 week). Right: Kaplan–Meier survival analysis comparing male (*n* = 10) and female (*n* = 10) mice after 344SQ-RFP cells injection. Statistical significance was determined using the log-rank test (*p* < 0.05). (**B**) Representative morphological images of lung and brain tissues from control, 6-week, and 10-week groups on a yellow background, showing visible changes. Arrows indicate regions of metastatic lesions. (**C**) RFP fluorescence imaging of lung and brain tissues at 6- and 10-weeks post-injection, revealing tumor progression through RFP-positive signals. (**D**) Representative H&E staining of brain sections at 6- and 10-weeks showing metastatic lesions at different magnifications: whole brain section (×10, scale bar = 2 mm) with arrow indicating metastatic region, low magnification of metastatic region (×100, scale bar = 200 μm), medium magnification showing tumor infiltration (×200, scale bar = 100 μm), and high magnification detailing metastatic cells (×400, scale bar = 50 μm).

To establish a lung cancer brain metastasis model, we subcutaneously injected 344SQ-RFP cells into the flanks of 8-week-old mice and monitored disease progression over 10 weeks (Figure 1A). The experimental timeline included two key analysis points: assessment of lung cancer at 6 weeks and brain metastasis at 10 weeks post-injection. Survival analysis revealed significant sex-dependent differences, with female mice demonstrating markedly better survival compared to male mice (*p* < 0.05). While male mice showed a rapid decline with a median survival of approximately 5 weeks, female mice exhibited a median survival of 8.5 weeks, with the last surviving female mouse reaching 11 weeks post-injection.

The temporal progression of cancer development was evaluated through direct visualization and fluorescence imaging of lung and brain tissues (Figure 1B,C). At 6 weeks post-injection, the lungs exhibited visible tumor nodules, clearly distinguishable from control tissues (Figure 1B). These nodules were confirmed through RFP fluorescence imaging. At 6 weeks, while metastasis was observed in key organs, including the lungs, spleen, and liver, with notably smaller tumor sizes compared to the 10-week group, brain tissues showed no metastatic changes with normal brain histology (Figure 1B and Supplementary Figure S1). By 10 weeks, RFP-positive signals indicated the presence of metastatic lesions in the brain (Figure 1C). Histological analysis through H&E staining showed normal brain histology in the 6-week group, whereas detailed confirmation of brain metastasis was observed in the 10-week group (Figure 1D). The whole brain section (×10) revealed the spatial distribution of metastatic lesions, which was further examined at increasing magnifications. Low magnification images (×100) showed the overall structure of the metastatic region, while medium magnification (×200) demonstrated the infiltration pattern of tumor cells. At high magnification (×400), detailed cellular morphology of metastatic cells within the brain parenchyma was clearly visible, characterized by distinctive morphological features and tissue organization patterns.

### 3.2. Characterization and Validation of Isolated Serum Exosomes

**Figure 2:**
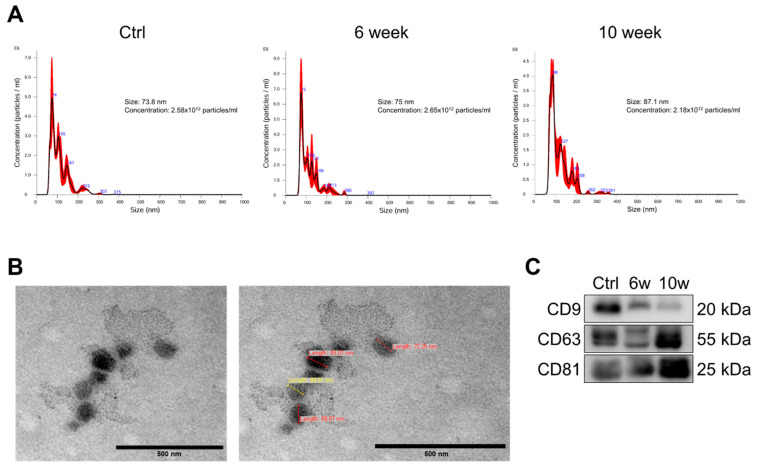
Characterization of isolated serum-derived exosomes. (**A**) Nanoparticle tracking analysis (NTA) showing size distribution and concentration of isolated exosomes in control, 6-week, and 10-week groups. (**B**) Representative transmission electron microscopy (TEM) image of isolated exosomes showing typical cup-shaped morphology. Scale bar = 0.5 μm. (**C**) Western blot analysis of exosomal marker proteins (CD9, CD63, and CD81) in control, 6-week, and 10-week groups. Equal amounts of protein (25 μg) were loaded for each sample.

To investigate the temporal changes in exosomal cargo during the progression from primary tumor to brain metastasis, exosomes were isolated from serum obtained from mice at 0-, 6-, or 10-weeks post-injection of 344SQ-RFP cells. Nanoparticle tracking analysis (NTA) revealed that the isolated exosomes exhibited mean diameters of 73.8 nm, 75 nm, and 87.1 nm for control, 6-week, and 10-week groups, respectively. The particle concentrations were 2.58 × 10^12^ particles/mL, 2.65 × 10^12^ particles/mL, and 2.18 × 10^12^ particles/mL for control, 6-week, and 10-week groups, respectively (Figure 2A). Transmission electron microscopy (TEM) imaging confirmed the typical cup-shaped morphology characteristic of exosomes (Figure 2B). The presence of canonical exosomal markers CD9, CD63, and CD81 was confirmed by immunoblotting, validating the successful isolation of exosomes from all experimental groups (Figure 2C). These results collectively demonstrate that the isolated vesicles from a lung cancer brain metastasis mouse model possess the characteristic features of exosomes in terms of size distribution, morphology, and specific marker expression. These isolated serum-derived exosomes of each experimental group were evaluated by RNA sequencing and proteomic analysis.

### 3.3. Comparison of miRNA Profiling and Bioinformatics Analysis of EV-Derived miRNAs Among the Control, 6w, and 10w Groups

**Figure 3:**
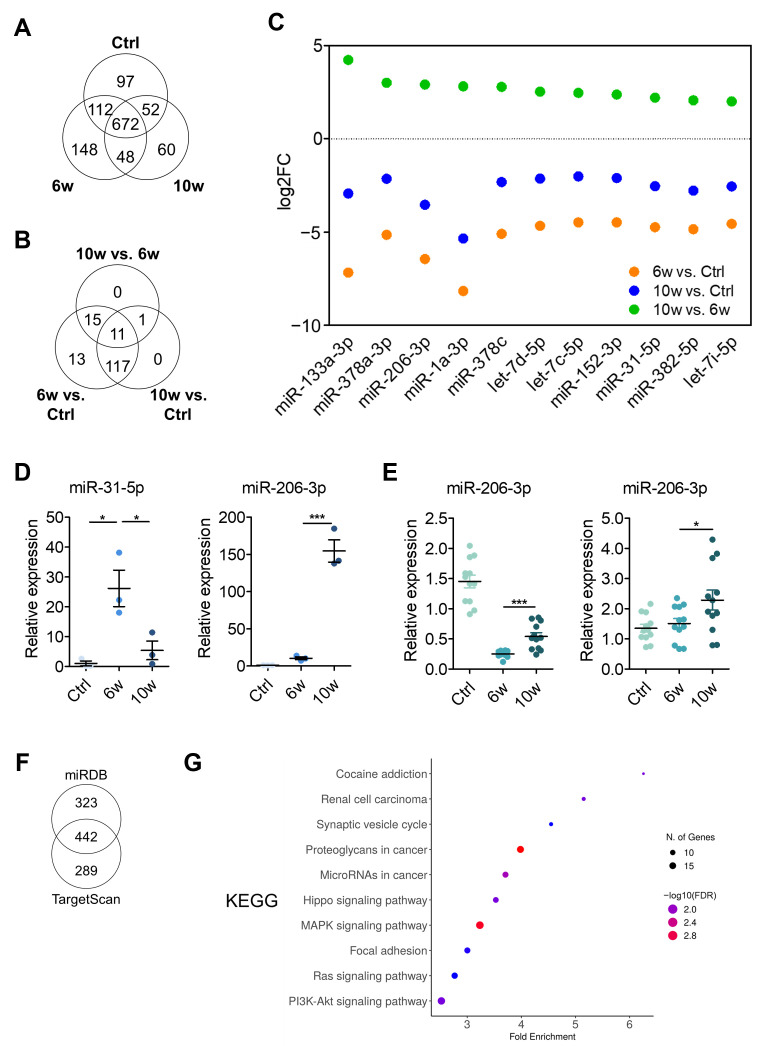
Comprehensive analysis of exosomal miRNAs expression profiles during cancer progression. (**A**) Venn diagram showing the number of mature miRNAs commonly and uniquely expressed in control, 6-week, and 10-week groups. (**B**) Venn diagram displaying the overlap of differentially expressed miRNAs (log2 fold change > 2) among different group comparisons. (**C**) Scatter plot of log2 fold changes for 11 commonly differentially expressed miRNAs across the three comparisons. Orange, blue, and green dots represent 6w vs. Control, 10w vs. Control, and 10w vs. 6w comparisons, respectively. (**D**) RT-qPCR validation of miR-31-5p and miR-206-3p expression in serum-derived exosomes from each group. Expression was normalized to miR-16-5p. Data are presented as mean ± SEM from three independent experiments. * *p* < 0.05, *** *p* < 0.001. (**E**) RT-qPCR analysis of miR-206-3p expression in lung (**left**) and brain (**right**) tissues. Expression was normalized to *RNU6B*. * *p* < 0.05, *** *p* < 0.001. (**F**) Venn diagram showing the overlap of predicted miR-206-3p target genes between the miRDB and TargetScan databases. (**G**) KEGG pathway enrichment analysis of miR-206-3p target genes.

To investigate transcriptomic differences in exosomes during cancer progression, we performed small RNA sequencing on exosomes isolated from pooled samples of each group. RNA sequencing of exosomes isolated from pooled samples identified 2099 miRNAs, including 1189 mature miRNAs. Of these, 672 mature miRNAs were commonly expressed across all groups (Figure 3A). After applying a filtering criterion of >100 read counts to minimize bias from low-expressing miRNAs, 160 miRNAs were selected for differential expression analysis.

Using a threshold of log2 fold change > 2, we identified differentially expressed miRNAs across different group comparisons (Figure 3B). The analysis revealed 156 differentially expressed miRNAs in the 6-week versus control comparison, and 129 miRNAs in the 10-week versus control comparison. The comparison between the 10-week and 6-week groups yielded 27 differentially expressed miRNAs. Notably, Venn diagram analysis identified 11 miRNAs that were consistently differentially expressed across all comparisons. Analysis of expression patterns revealed an intriguing trend: all 11 miRNAs showed decreased expression in both the 6-week and 10-week groups compared to the control group, but demonstrated increased expression in the 10-week group relative to the 6-week group (Figure 3C). This pattern suggests potential stage-specific roles of these miRNAs during cancer progression.

From these 11 miRNAs, we selected miR-31-5p and miR-206-3p as candidates for further validation, based on their contrasting functional roles in lung cancer progression. miR-31-5p has been reported to promote tumorigenesis, whereas miR-206-3p is known to act as a metastasis suppressor [29,30]. To validate these findings, we performed RT-qPCR analysis to examine their expression in serum-derived exosomes across the control, 6-week, and 10-week groups (Figure 3D). miR-31-5p expression exhibited significant upregulation at 6 weeks, followed by a decrease at 10 weeks. In contrast, miR-206-3p was relatively low until 6 weeks post-injection when lung metastasis began to be observed, but demonstrated marked elevation at 10 weeks. Notably, the expression pattern of miR-31-5p observed in RT-qPCR was inconsistent with the RNA-seq results, which showed upregulation at 10 weeks. Due to this discrepancy between sequencing and validation data, we excluded miR-31-5p from further consideration as a potential biomarker candidate.

To gain further insight into the role of miR-206-3p in organ-specific metastasis, we performed RT-qPCR analysis on brain and lung tissues from the 6-week and 10-week groups (Figure 3E). In the brain, a significant upregulation of miR-206-3p was observed specifically in the 10-week group, coinciding with the onset of brain metastasis. Conversely, in the lung, miR-206-3p levels were consistently downregulated compared to control mice and 6-week and 10-week groups, suggesting its potential involvement in the later stage of cancer metastasis and progression.

To elucidate the potential target genes of miR-206-3p in brain metastasis, we performed target prediction analysis using a combination of the databases miRDB and TargetScan. We uncovered 224 miR-206-3p-target gene pairs (Figure 3F). KEGG pathway analysis of the predicted target genes revealed significant enrichment in several cancer-related pathways (Figure 3G), including Hippo, MAPK, Ras, and PI3K-Akt signaling pathways. Additionally, the analysis identified enrichment in focal adhesion and proteoglycans in cancer pathways, suggesting a potential regulatory role of miR-206-3p in these processes. For comparison, KEGG pathway analysis was also performed for miR-31-5p using the target genes identified from the miRDB and TargetScan databases. A total of 177 common target genes were identified, and pathway analysis revealed significant enrichment in axon guidance and tight junction pathways (Supplementary Figure S2). These results revealed distinct temporal patterns of miRNA expression in serum-derived exosomes during cancer progression, from primary tumor to brain metastasis, particularly highlighting the expression of these 11 shared miRNAs.

### 3.4. Comparison of Protein Profiling and Bioinformatics Analysis of EV-Derived Proteins Among the Control, 6w, and 10w Groups

**Figure 4:**
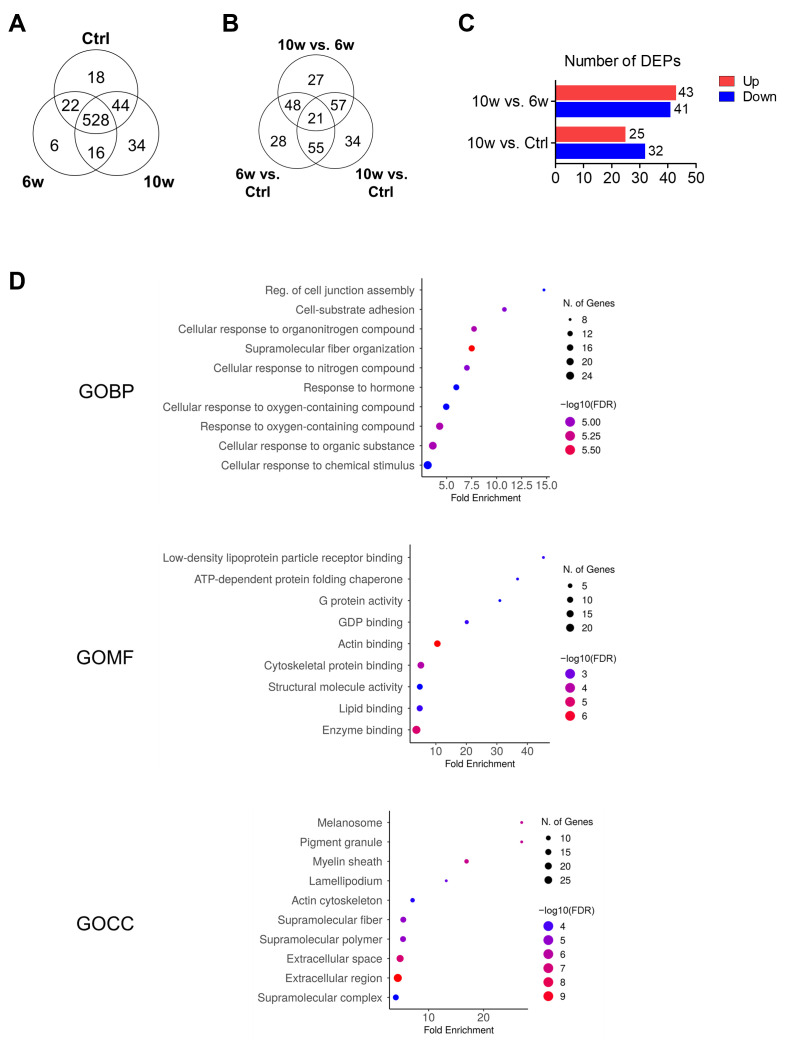
Proteomic analysis of differentially expressed exosomal proteins. (**A**) Venn diagram showing the distribution of identified proteins across control, 6-week, and 10-week groups. (**B**) Distribution of differentially expressed proteins (DEPs) among group comparisons. Proteins showing more than 2-fold change in abundance were considered differentially expressed (upregulated: fold change > 2, downregulated: fold change < 0.5). (**C**) Number of up- and downregulated DEPs in the comparison of 10-week vs. 6-week and 10-week vs. control groups. (**D**) Gene Ontology (GO) enrichment analysis of 77 upregulated proteins in the 10-week group, categorized by biological process (GOBP), molecular function (GOMF), and cellular component (GOCC).

We next performed proteomic analysis using LC-MS/MS to investigate temporal changes in the expression level of exosomal proteins during the progression from primary tumor to brain metastasis. A total of 668 proteins were identified, with 528 proteins shared across all groups. The remaining proteins showed group-specific expression patterns: 18 proteins were unique to the control group, 6 proteins were exclusive to the 6-week group, and 34 proteins were found only in the 10-week group (Figure 4A).

Analysis of differentially expressed proteins (DEPs) among the 528 shared proteins revealed distinct patterns between groups using a 2-fold change threshold. The comparison between the 6-week and control groups identified 152 DEPs, with 30 upregulated and 122 downregulated proteins. Between the 10-week and control groups, 167 DEPs were identified (69 upregulated, 98 downregulated). The comparison between 10-week and 6-week groups revealed 153 DEPs, comprising 96 upregulated and 57 downregulated proteins (Figure 4B). Based on our hypothesis that exosomes from the 10-week group would contain proteins specifically associated with cancer progression and metastasis, we focused our analysis on proteins showing increased expression in the 10-week group comparisons. In the comparison between groups, we identified 84 proteins (comprising 27 proteins unique to the 10-week vs. 6-week comparison and 57 proteins overlapping between 10-week vs. 6-week and 10-week vs. control comparisons) with altered expression patterns (Figure 4B,C). For further analysis, we focused on a total of 77 proteins that were associated with the brain metastasis stage, consisting of 34 proteins uniquely expressed in the 10-week group (Figure 4A) and 43 proteins showing increased expression in the 10-week vs. 6-week group comparison.

Gene Ontology (GO) analysis of these 77 proteins revealed their functional significance across three categories (Figure 4D). In terms of biological processes (GOBP), the proteins were significantly enriched in pathways related to regulation of cell junction assembly, cell–substrate adhesion, cellular response to organonitrogen compounds, and supramolecular fiber organization. Molecular function analysis (GOMF) showed enrichment in low-density lipoprotein particle receptor binding, ATP-dependent protein folding chaperone, G protein activity, and GDP binding. Cellular component analysis (GOCC) indicated significant association with melanosome, pigment granule, myelin sheath, lamellipodium, and actin cytoskeleton.

**Figure 5:**
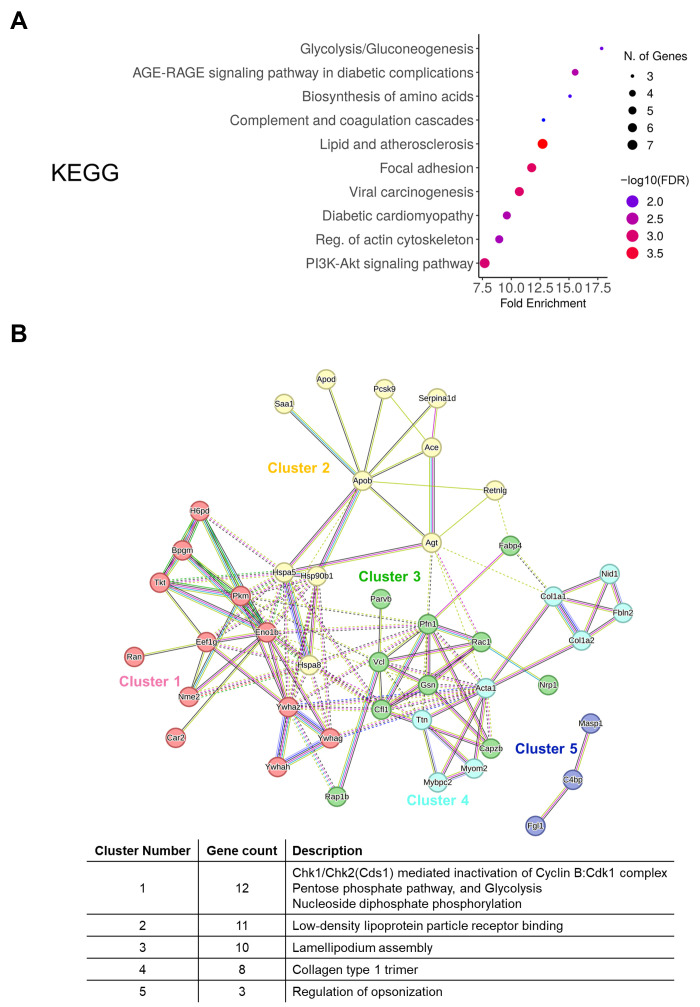
Pathway and network analysis of differentially expressed proteins. (**A**) KEGG pathway enrichment analysis of 77 proteins upregulated in the 10-week group. Color intensity indicates significance levels, and circle size represents gene count. (**B**) STRING-based protein–protein interaction network analysis showing five functional clusters. The table shows cluster descriptions and gene counts. Different colors represent distinct function clusters, and connecting lines indicate protein–protein interactions based on STRING database confidence scores.

Also, in the KEGG pathway analysis, the proteins were mainly involved in glycolysis/gluconeogenesis, the AGE-RAGE signaling pathway in diabetic complications, biosynthesis of amino acids, and complement and coagulation cascades (Figure 5A). We then analyzed the protein–protein interaction (PPI) network of DEPs using the STRING database. This analysis identified five distinct functional clusters: Cluster 1 (12 genes) was associated with Chk1/Chk2-mediated inactivation of the Cyclin B/Cdk1 complex, pentose phosphate pathway, and glycolysis; Cluster 2 (11 genes) with low-density lipoprotein particle receptor binding; Cluster 3 (10 genes) with lamellipodium assembly; Cluster 4 (8 genes) with collagen type 1 trimer; and Cluster 5 (3 genes) with regulation of opsonization (Figure 5B). Quantitative analysis of inter-cluster interactions revealed that Cluster 3, which includes vinculin (VCL), showed significant connectivity with Clusters 1, 2, and 4 (Supplementary Figure S3). VCL was among the proteins contributing to these inter-cluster connections, suggesting a potential role in linking cytoskeletal organization with adhesion-related pathways. Collectively, our proteomic analysis identified a subset of 77 proteins that were specifically upregulated in or unique to the metastatic stage, with significant involvement in cell adhesion, metabolic pathways, and cellular organization. These findings suggest potential mechanistic pathways involved in the progression of brain metastasis.

### 3.5. Validation of VCL Expression in Tissues and Exosomes

**Figure 6:**
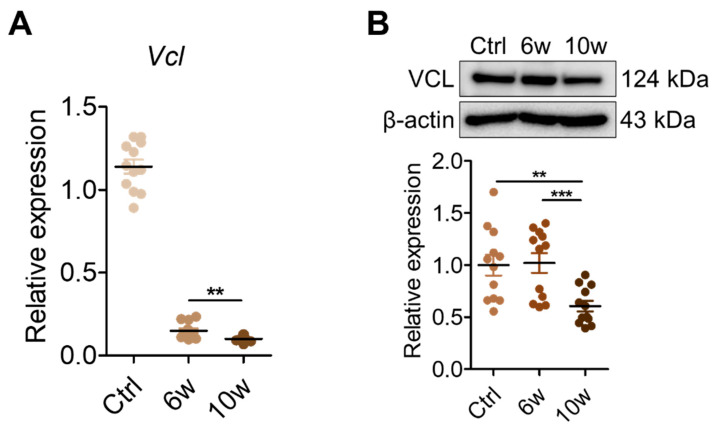

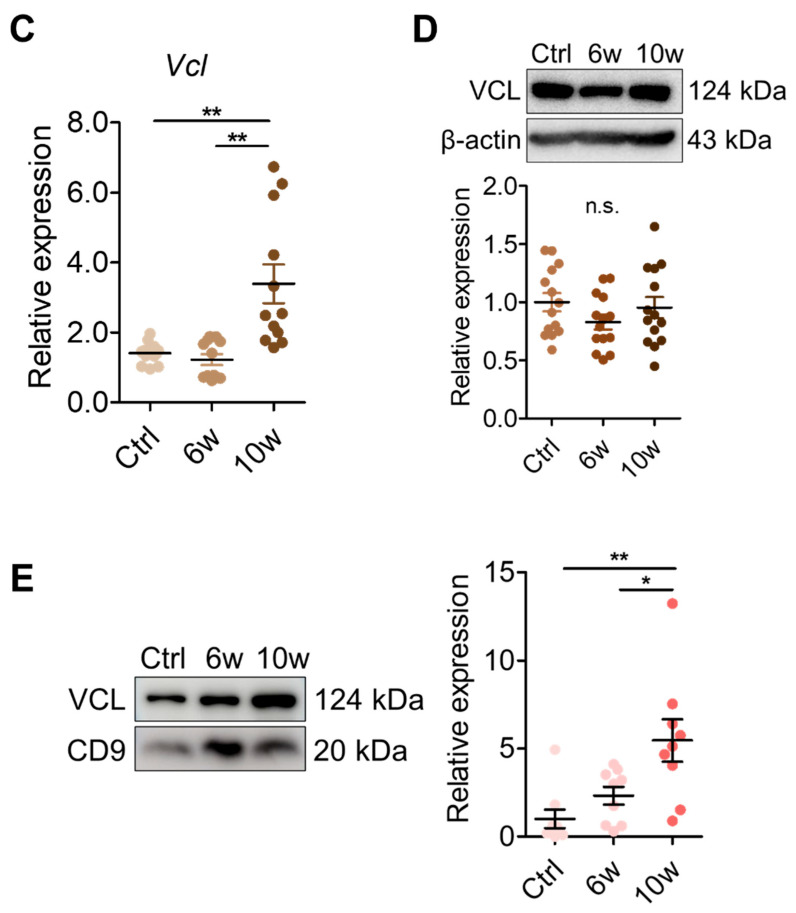
Assessment of VCL expression in lung, brain, and serum-derived exosomes. (**A**) RT-qPCR analysis of Vcl mRNA expression in lung tissues from control, 6-week, and 10-week groups. Expression was normalized to Gapdh. Data represent mean ± SEM from three independent experiments. (**B**) Western blot analysis of VCL protein in lung tissues. Band intensities were normalized to β-actin. (**C**) RT-qPCR analysis of Vcl mRNA expression in brain tissues from control, 6-week, and 10-week groups. Expression was normalized to Gapdh. Data represent mean ± SEM from three independent experiments. (**D**) Western blot analysis of VCL protein in brain tissues. Band intensities were normalized to β-actin. (**E**) Western blot analysis of VCL in serum-derived exosomes. CD9 was used as an exosome marker and loading control. Statistical significance is indicated as follows: * *p* < 0.05, ** *p* < 0.01, *** *p* < 0.001, and n.s. (not significant).

**Figure 7:**
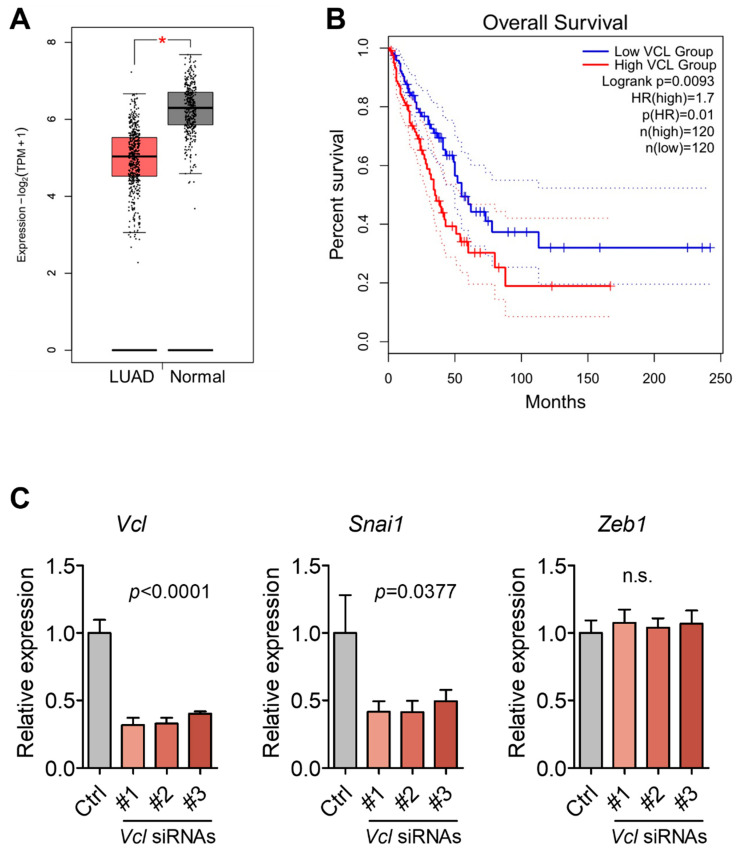
Clinical relevance of VCL expression and its regulatory effect on mesenchymal markers. (**A**) VCL expression levels in lung adenocarcinoma (LUAD) tumor tissues compared to normal tissues were analyzed using the GEPIA2 database. * *p* < 0.05. (**B**) Kaplan–Meier survival analysis of LUAD patients stratified by VCL expression quartiles. Log-rank *p* = 0.0093; HR = 1.7. Dotted lines represent 95% confidence intervals. (**C**) Expression levels of mesenchymal markers following siRNA-mediated knockdown of *Vcl* in 344SQ cells. “#” denotes different siRNA constructs targeting *Vcl*. Differences among groups were assessed by one-way ANOVA followed by Tukey’s post hoc test. Data are presented as mean ± SEM from three independent experiments. n.s., not significant.

Given VCL’s role in linking integrins to the actin cytoskeleton and its known association with metastasis [31,32,33], we examined its expression levels in lung and brain tissues, as well as serum-derived exosomes. In lung tissues, *Vcl* mRNA expression was significantly decreased in the 10-week group compared to the control and 6-week groups (Figure 6A). Immunoblot analysis similarly showed a reduction in VCL protein levels at 10 weeks (Figure 6B). In the brain tissues, *Vcl* mRNA levels were increased in the 10-week group (Figure 6C), while protein levels remained comparable across groups (Figure 6D). In contrast, serum-derived exosomes from the 10-week group showed a marked increase in VCL protein levels compared to control and 6-week groups, as determined by immunoblotting (Figure 6E). These findings reveal tissue-specific regulation of VCL expression during cancer progression, and a pronounced elevation of exosomal VCL levels at the stage of brain metastasis, suggesting its potential relevance to metastatic progression. Consistent with our findings, analysis using the GEPIA2 database, which includes RNA-seq data from The Cancer Genome Atlas (TCGA) lung adenocarcinoma (LUAD) samples (483 tumor and 347 normal tissues), revealed decreased VCL expression in LUAD tissues compared to normal tissues (Figure 7A). To evaluate the clinical relevance of VCL expression, we performed Kaplan–Meier survival analysis by stratifying patients into quartiles based on VCL expression levels. Patients in the top quartile (Q4) exhibited significantly worse overall survival compared to those in the bottom quartile (Q1) (Figure 7B). Furthermore, *Vcl* knockdown in 344SQ lung cancer cells selectively decreased *Snai1* expression without affecting *Zeb1*, another mesenchymal marker (Figure 7C). These results suggest a potential regulatory link between *Vcl* and *Snai1*, rather than a broad effect on EMT-regulated gene expression.

## 4. Discussion

Brain metastasis is most commonly observed in lung cancer patients and is associated with poor prognosis due to late detection. Our study aimed to identify potential biomarkers for the detection of brain metastasis by comprehensively analyzing exosomal contents during disease progression. Through integrated multi-omics analysis, we identified miR-206-3p and VCL as promising biomarkers that show significant changes during brain metastasis development.

The standard of care for diagnosing brain metastasis relies heavily on advanced neuroimaging modalities, including MRI, CT, and PET scans [34]. While these imaging techniques provide high-resolution and accurate diagnostics, they are limited in their ability to detect metastatic lesions at an early stage, when therapeutic interventions may be more effective [34,35]. Additionally, the cumulative radiation exposure from repeated CT scans raises long-term safety concerns for patients requiring frequent monitoring [36]. In contrast, liquid biopsy using exosomal biomarkers offers several advantages over conventional neuroimaging approaches. Liquid biopsy enables minimally invasive sampling, allowing for more frequent monitoring of disease progression in a cost-effective manner [37]. Importantly, liquid biopsy holds the potential for detecting metastatic processes before they become apparent through conventional imaging techniques, thereby enabling earlier therapeutic intervention and improved patient outcomes.

The selection of factors showing alterations at 10 weeks as candidate biomarkers for late metastasis and brain colonization was driven by our systematic temporal analysis. Our mouse model demonstrated a distinct pattern of metastatic progression, where metastases to peripheral organs (lungs, liver, spleen, and kidneys) were detected at 6 weeks post-injection, while brain metastases emerged uniquely at 10 weeks (Supplementary Figure S1). This temporal segregation enabled us to conduct targeted screening by excluding factors with similar expression changes at week 6, thereby establishing a robust framework for identifying brain metastasis-specific biomarkers. Based on this systematic approach, we identified several promising candidates, with miR-206-3p and VCL showing particularly significant alterations during brain metastasis development.

Our findings highlight exosomal miR-206-3p as a potential biomarker for brain metastasis in lung cancer. Its expression remained low during early tumor development but was significantly elevated in exosomes at the metastatic stage. Notably, miR-206-3p was downregulated in lung tissues but upregulated in brain tissues, suggesting a role in brain-specific metastasis. Although miR-206-3p has been reported as a tumor suppressor in lung cancer, we observed its downregulation in lung tissues in both the 6-week and 10-week groups compared to controls. However, its expression was relatively increased in the 10-week group, particularly in serum-derived exosomes and brain tissues. These findings suggest that miR-206-3p may exhibit stage- or tissue-specific patterns during metastatic progression. While the underlying mechanisms remain unclear, this observation raises the possibility of a context-dependent role for miR-206-3p that warrants further investigation. Along with similar findings in the blood of lung cancer patients [38], these results suggest that its role may vary depending on tumor stage and tissue type. Such dynamic regulation implies that miR-206-3p may contribute to metastatic adaptation, rather than serving solely as a tumor suppressor.

Our proteomic analysis identified 77 proteins that were specifically upregulated or uniquely present in the 10-week group. GO term enrichment analysis revealed that these proteins were significantly associated with biological processes such as cell junction assembly, cell–substrate adhesion, and supramolecular fiber organization. These features are critical for metastatic dissemination and suggest that changes in adhesion and cytoskeletal dynamics are key events in brain metastasis development. Interestingly, cellular component analysis also highlighted terms related to the myelin sheath and lamellipodium, structures relevant for neural interactions and cellular motility, respectively, pointing to the adaptation of metastatic cells to the brain microenvironment [39,40]. Given these findings, we focused on VCL, a key cytoskeletal protein that links integrin adhesion complexes to the actin cytoskeleton. VCL was among the significantly upregulated proteins in our exosome proteome. In agreement with its known involvement in tumor aggressiveness and invasion [41], our data demonstrated that *Vcl* mRNA and protein expression levels were decreased in lung tissues at 10 weeks, while exosomal VCL protein levels were significantly increased. This inverse relationship between tissue and exosomal VCL levels may reflect a shift in protein distribution during metastatic progression, the biological implications of which warrant further investigation. Moreover, patient data analysis revealed that high VCL expression is associated with poor overall survival in LUAD (Figure 7B), supporting its clinical relevance. Although knockdown of *Vcl* did not consistently affect multiple EMT markers (Figure 7C), we observed a significant reduction in *Snai1*, a key regulator of EMT, suggesting a partial involvement in metastatic phenotype regulation. Together, these findings suggest that exosomal VCL may be actively regulated during metastasis and play a role in reprogramming tumor cell communication, thereby facilitating adaptation to the brain microenvironment.

This study has several limitations. First, the use of a single murine lung cancer cell line (344SQ-RFP) limits the generalizability of our findings. Although this model was selected for its well-characterized metastatic potential and previous evidence supporting its clinical relevance—including the predictive value of Tspan8 in human NSCLC—it remains important to validate the observed biomarkers in additional cell lines and patient-derived models [42]. Second, the cellular origin of exosomal VCL remains unclear. In our experimental model, we observed increased VCL mRNA levels in metastatic brain tissue as well as elevated VCL levels in circulating exosomes. However, we cannot definitively determine whether the observed exosomal VCL originates from tumor cells, stromal cells, or immune cells within the brain metastatic microenvironment. To address this, future studies employing single-cell transcriptomics, lineage tracing, or vesicle profiling will be needed to identify the precise source of exosomal VCL. Third, due to the consistently poor survival of male mice before reaching the 10-week endpoint, all of our experiments were conducted using female-derived samples. While male mice were excluded for this reason, future studies employing sex-balanced designs and optimized model systems will be necessary to confirm the generalizability of our findings.

While our current data do not establish a direct mechanistic link between exosomal VCL and brain metastasis, several observations suggest a potential association. Notably, exosomal VCL expression was not elevated at 6 weeks, when brain metastasis was absent, but showed a marked increase at 10 weeks, coinciding with the onset of brain metastasis. Furthermore, tissue-level analysis at 10 weeks revealed a selective increase in *Vcl* mRNA expression in brain tissues, whereas levels in the lung and liver remained unchanged or were reduced. These findings raise the possibility that exosomal VCL upregulation may reflect brain-specific metastatic progression rather than a general response to systemic metastasis.

In conclusion, this study presents a lung cancer brain metastasis mouse model and investigates temporal alterations in exosomal components during cancer progression. Among the identified molecules, miR-206-3p and VCL exhibited dynamic and context-dependent expression patterns across tissues and serum-derived exosomes. It will be interesting to determine whether the contrasting expression of VCL in tissues and exosomes reflects selective sorting related to metastatic progression or merely mirrors cancer progression-associated changes. Given the growing interest in exosome-based biomarkers, our findings may provide the potential utility of exosomal miR-206-3p and VCL as diagnostic and prognostic tools in metastatic lung cancer.

## 5. Conclusions

In this study, we established a lung cancer brain metastasis mouse model and demonstrated the potential utility of serum-derived exosomes for monitoring brain metastasis development. Through comprehensive multi-omics analysis of exosomal contents, we identified miR-206-3p and VCL as promising molecular signatures associated with late-stage brain metastasis. Pathway analysis revealed that miR-206-3p is involved in critical cancer-related pathways, while VCL exhibited distinctive expression patterns between tissues and exosomes during metastasis progression. The use of a well-characterized animal model enabled us to trace molecular changes across defined stages of metastasis, thereby enhancing the translational relevance of our findings, with possible implications for clinical research. To enhance the clinical relevance of our findings, it will be important to validate the expression patterns of candidate biomarkers in patient-derived samples. If future studies identify common key factors between our animal model and clinical specimens, our model may serve as a valuable platform for elucidating the mechanisms of action, conducting functional studies, and investigating their roles in promoting brain metastatic progression.

## Data Availability

The data presented in this study are available on request from the corresponding author. The data are not publicly available due to institutional policy and ongoing related research.

## Supplementary Materials

The following supporting information can be downloaded at https://www.mdpi.com/article/10.3390/cancers17121929/s1: Supplementary Table S1. qRT-PCR primers used in this study. Supplementary Figure S1. Representative morphological images of peripheral organs from control, 6-week, and 10-week groups showing visible changes. Images of liver, spleen, and kidney tissues on a yellow background, showing the temporal progression of cancer development. Supplementary Figure S2. Analysis of miR-31-5p target genes and associated pathways. (A) Venn diagram showing overlap of predicted target genes for miR-31-5p between miRDB and TargetScan databases, identifying 177 common target genes. (B) KEGG pathway analysis of the 177 common target genes reveals significant enrichment in axon guidance and tight junction pathways. Supplementary Figure S3. Quantitative analysis of inter-cluster interactions in the protein–protein interaction network. The number of interactions and average combined scores between different clusters are shown. Combined scores range from 0 to 1, with higher scores indicating stronger confidence in the interaction.
