# Supplementary material for: Comprehensive Profiling of Serum Exosomes by a Multi-Omics Approach Reveals Potential Diagnostic Markers for Brain Metastasis in Lung Cancer
Source: Cancers (Basel). 2025 Jun 10;17(12):1929. doi: 10.3390/cancers17121929 (PMC12191069; doi:10.3390/cancers17121929)
Supplement: Supplementary file 1 [file cancers-17-01929-s001.zip › cancers-3630104-supplementary.pdf]

Supplementary Table S1. qRT-PCR primers used in this study.

Genes	Forward	Reverse
Gapdh	CATCACTGCCACCCAGAAGACTG	ATGCCAGTGAGCTTCCCGTTCAG
Vcl	CCTATCAAGCTGTTGGCAGTAGC	TGTGGCTCCAAGCCTTCCTGAA
Snai1	TGTCTGCACGACCTGTGGAAAG	CTTCACATCCGAGTGGGTTTGG
Zeb1	ATTCAGCTACTGTGAGCCCTGC	CATTCTGGTCCTCCACAGTGGA

Supplementary Figure S1. Representative morphological images of peripheral organs from control, 6-week, and 10-week groups showing visible changes. Images of liver, spleen, and kidney tissues on yellow background, showing temporal progression of cancer development.

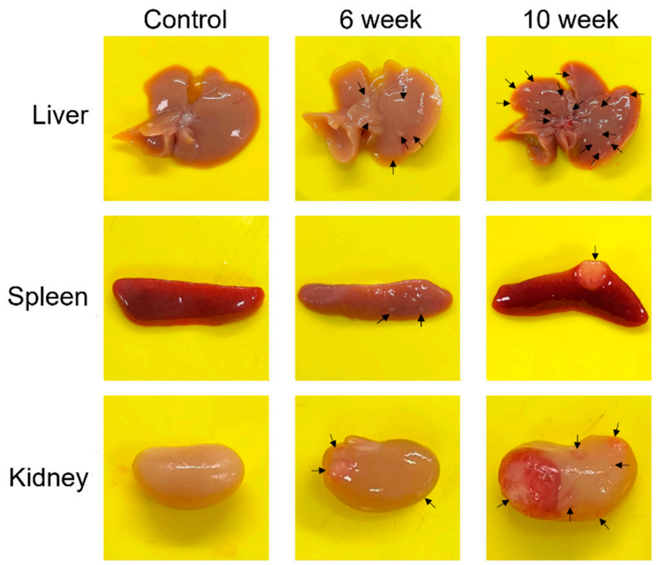

Supplementary Figure S2. Analysis of miR-31-5p target genes and associated pathways. (A) Venn diagram showing overlap of predicted target genes for miR-31-5p between miRDB and TargetScan databases, identifying 177 common target genes. (B) KEGG pathway analysis of the 177 common target genes reveals significant enrichment in axon guidance and tight junction pathways.

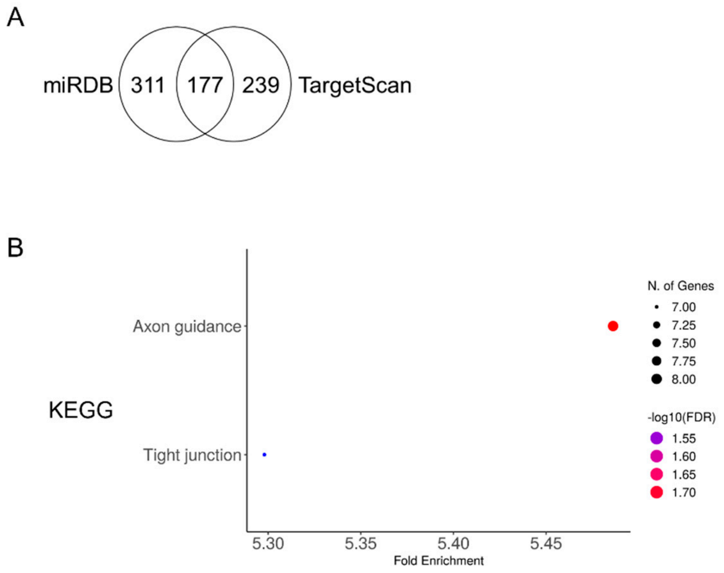

Supplementary Figure S3. Quantitative analysis of inter-cluster interactions in the protein-protein interaction network. The number of interactions and average combined scores between different clusters are shown. Combined scores range from 0 to 1, with higher scores indicating stronger confidence in the interaction.

Cluster	Cluster	Interactions	Average Score
1	2	19	0.549
1	3	8	0.575
1	4	2	0.449
2	3	5	0.558
2	4	4	0.469
3	4	8	0.569
